# A Simplified Procedure for Intrathecal Baclofen Catheter Replacement Through a Granulation Tunnel Using a 16-Gauge Peripheral Vein Catheter

**DOI:** 10.7759/cureus.77686

**Published:** 2025-01-19

**Authors:** Kotaro Kohara, Maegawa Tatsuya

**Affiliations:** 1 Department of Neurosurgery, Tokyo Women's Medical University, Tokyo, JPN; 2 Department of Spine Surgery, Kameda Medical Center, Kamogawa, JPN

**Keywords:** catheter replacement, granulation tunnel, intrathecal baclofen, outer sleeve, peripheral vein catheter

## Abstract

Intrathecal baclofen (ITB) therapy is an established surgical treatment for spasticity. Long-term management of ITB may necessitate catheter replacement, which typically requires a new dural puncture. Although rare, complications such as cerebrospinal fluid leakage, nerve injury, and hemorrhage can occur with a new dural puncture. We successfully replaced an intrathecal catheter of ITB through a granulation tunnel formed around the old catheter using a 16-gauge vein catheter, without the need for a new dural puncture. A 79-year-old man with spasticity resulting from cervical spinal cord infarction underwent ITB therapy and required intrathecal catheter replacement. During the replacement surgery, a 16-gauge indwelling peripheral vein catheter was inserted into the granulation tunnel around the old catheter, using the old catheter as a guide. After the old catheter was removed, the vein catheter remained in place, and a new intrathecal catheter was carefully introduced through the vein catheter. The catheter was successfully advanced into the subarachnoid space via the vein catheter and granulation tunnel, completing the procedure without any surgical complications. This technique can reduce the risks associated with dural puncture, lower radiation exposure, and decrease the overall surgical time.

## Introduction

Intrathecal baclofen (ITB) therapy is an established surgical treatment for spasticity and dystonia [1-4]. In the long-term management of ITB, catheter malfunctions, such as occlusion, disconnection, or breakage occur in 15.1-17.6% of cases, and catheter replacement may be necessary [5-8].

The replacement procedure for an intrathecal catheter typically requires a new dural puncture. Although catheter replacement is usually straightforward [8], complications such as cerebrospinal fluid (CSF) leak, nerve injury, and intra-/extradural hemorrhage can occur during catheter replacement surgery due to a new dural puncture. Although CSF leak occurs in 4.9-24.9% of cases, and reoperation is a risk factor [5,9,10], no detailed reports address surgical techniques to prevent such complications during ITB catheter replacement.

When a foreign material is implanted in the human body, granulation tissue encases it over time. Consequently, a granulation tunnel develops around spinal cord stimulation (SCS) leads, deep brain stimulation leads, or ITB catheters. In SCS, technical reports have described the replacement of epidural leads through granulation tunnels formed around the old leads, avoiding the need for a new puncture [11,12].

Herein, we describe a surgical technique in which an intrathecal catheter for ITB was successfully replaced through a granulation tunnel formed around the old catheter using a 16-gauge indwelling peripheral venous catheter without a new dural puncture.

## Case presentation

The patient was a 79-year-old man with a medical history of atrial fibrillation and type 2 diabetes mellitus. He experienced a cervical spinal cord infarction five years earlier, resulting in truncal tightening pain and a squeezing sensation. He has been managing these symptoms by taking tramadol, acetaminophen, pregabalin, and tizanidine for neuropathic pain and spasticity. He exhibited a spastic gait, a tingling sensation in the lower extremities, and increased deep tendon reflexes in both the upper and lower extremities. The modified Ashworth scale score was 1+. An ITB trial with 50 μg baclofen provided relief from the truncal tightening, leading to the initiation of ITB therapy.

During the initial surgery, an Ascenda catheter (model 8781; Medtronic, Minneapolis, MN, US) was inserted at the L3/4 vertebral level into the subarachnoid space, with the catheter tip placed at the T8 vertebral level, corresponding to the tightest area within the dermatome. A SynchroMed II pump (Medtronic) was implanted subcutaneously in the abdomen.

Following the first surgery, the pump rate was gradually increased and the spasticity of the lower extremities was relieved, but a total baclofen dose of 180 μg/day did not alleviate the truncal symptoms. Further dose increases were limited by side effects, including sleepiness and weakness in the lower extremities. Despite adjustments using the flex mode, the situation remained unresolved. No abnormalities were found in the reservoir residual volume. Nine months after the initial surgery, we decided to replace the intrathecal catheter and reposition the catheter tip from the T8 to the upper thoracic vertebral level.

Operative procedure

**Figure 1:**
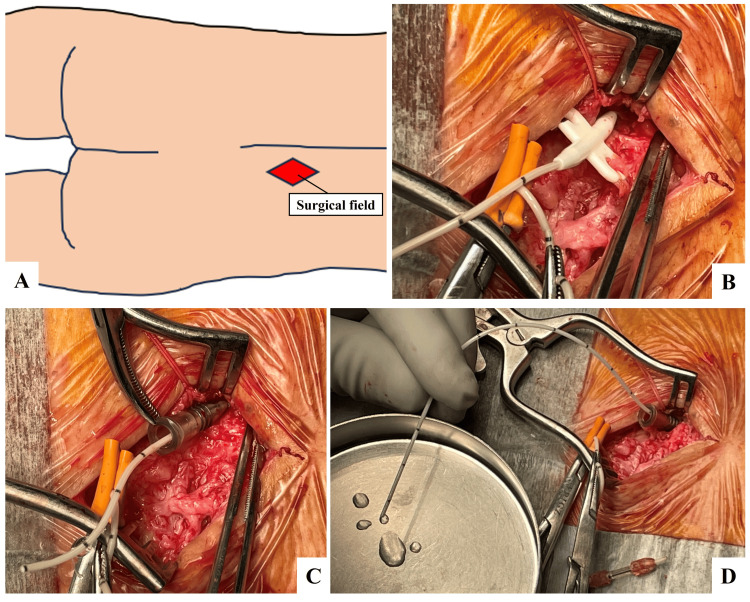
Intraoperative photographs of surgical field A schema of the surgical field(A), an anchor device fixing the spinal segment of an Ascenda catheter to the thoracolumbar fascia (B), and a 16-gauge indwelling peripheral vein catheter inserted into the granulation tunnel formed around the old catheter by using the old catheter as a guide (C) Cerebrospinal fluid backflow was observed from the catheter throughout the procedure(D). Panel A was created by the authors.

**Figure 2:**
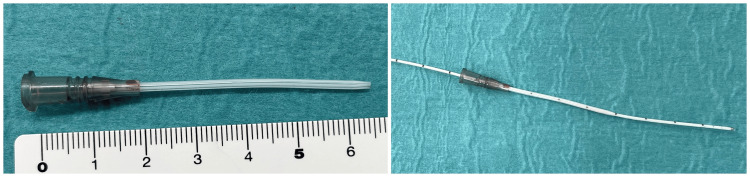
A 16-gauge indwelling peripheral vein catheter and an Ascenda catheter A 16-gauge indwelling peripheral vein catheter with a lumen diameter that is just large enough for the Ascenda catheter to pass through

**Figure 3:**
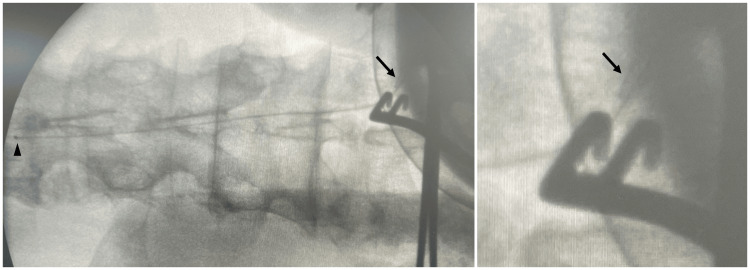
Intraoperative fluoroscopy Intraoperative fluoroscopy showing the tip of a 16-gauge indwelling peripheral vein catheter (arrow) and the tip of the intrathecal catheter (arrowhead)

The catheter replacement surgery was performed under general anesthesia, with the patient in a prone position. We reopened the previous skin incision in the lumbar region and meticulously traced the catheter running subcutaneously to the thoracolumbar fascial penetration point, where we confirmed the presence of an anchor device securing the catheter to the fascia (Figure 1). The anchor was carefully detached from the fascia, the intrathecal catheter was withdrawn from the spinal canal, and the catheter was cut just distal to the anchor. A 16-gauge indwelling peripheral vein catheter (Surflo®, Terumo Co., Tokyo, Japan) has a lumen diameter that is just large enough for an Ascenda catheter to pass through (Figure 2). Using the old catheter as a guide, the vein catheter was inserted into the granulation tunnel formed around the old catheter (Figure 1). We removed the old catheter, leaving the vein catheter within the granulation tunnel and confirming the CSF backflow from the vein catheter, and a new Ascenda catheter was carefully inserted into the vein catheter. The new catheter was smoothly advanced into the subarachnoid space, confirmed using intraoperative fluoroscopy (Figure 3), and the catheter tip was positioned at the T1 vertebral level. CSF backflow was observed from the catheter (Figure 1). We removed the vein catheter, secured the Ascenda catheter to the fascia using an anchor, connected the entire ITB system, and reimplanted it subcutaneously.

Postoperative course

The truncal tightening sensation was relieved with a lower dose of baclofen, without any surgical complications.

## Discussion

We successfully replaced the intrathecal catheter for ITB therapy with a 16-gauge indwelling peripheral vein catheter inserted into a granulation tunnel formed around the old catheter without a new dural puncture. The vein catheter maintained access to the granulation tunnel and helped the smooth replacement of the Ascenda catheter as an outer sleeve.

Catheter replacement surgery may be required for catheter malfunctions [6], and a revision procedure is a risk factor for a CSF leak and is particularly common in pediatric patients [10]. Although intrathecal catheter replacement through a new dural puncture is usually simple, it is challenging in patients with strong scoliosis or vertebral deformity, and computed tomography (CT) image-guided procedures have been reported for such cases [13]. However, our method may replace an intrathecal catheter simply and smoothly without a specific technique or a new dural puncture. This can reduce the risk of not only CSF leakage but also nerve injury and hemorrhage. Furthermore, total surgical time may be shortened.

Granulation formed around artificial materials can develop several months after the implantation [14]. In SCS epidural lead replacement, a method similar to the one described above, fibrosis surrounding the old lead that formed after some weeks or months guided the reinsertion of a new SCS epidural lead [11,12]. Therefore, we speculated that the method would become feasible several months after the previous catheter implantation.

If the accompanying puncture needle with an Ascenda catheter kit (model 8781) is used as a guide, the catheter can get caught on the edge of the needle and break when pulling out the old catheter. Therefore, we used a vein catheter as a guide to avoid breaking it. Because the distance between the thoracolumbar fascia and the dura mater was >50 mm on CT, the tip of the vein catheter did not reach the subarachnoid space. However, because the granulation tunnel was firmly formed, this procedure was feasible. The vein catheter does not need to be sufficiently long to reach the subarachnoid space but needs to be inserted into a granulation tunnel.

The InDura catheter (Medtronic), which has a larger diameter than the Ascenda catheter, was used previously. Although this method does not directly replace an InDura catheter with an Ascenda catheter because of its larger diameter, if a 16-gauge vein catheter could be inserted into a granulation tunnel immediately after removing the old InDura catheter, a similar exchange from an InDura to an Ascenda catheter could be possible.

In either case, the procedure should be performed to confirm CSF backflow through the catheter and the position of the catheter tip with intraoperative fluoroscopy; preparing a new puncture is also recommended. If there is resistance at the surgeon’s fingertips while inserting an intrathecal catheter through a vein catheter into the subarachnoid space, the procedure should be stopped and a new puncture should be performed to avoid the risk of damaging the nerves and surrounding tissue and migration of the catheter.

## Conclusions

We successfully replaced an intrathecal catheter for ITB therapy using a 16-gauge indwelling peripheral vein catheter inserted into a granulation tunnel formed around the old catheter, without the need for a new dural puncture. This technique can reduce the risks associated with dural puncture. Further investigation is required to determine whether this procedure can be successfully replicated in other cases, and it may be better to get adequate approval at each hospital as the use of venous catheters for the method is an off-label use.
